# USP42 enhances homologous recombination repair by promoting R-loop resolution with a DNA–RNA helicase DHX9

**DOI:** 10.1038/s41389-020-00244-4

**Published:** 2020-06-15

**Authors:** Misaki Matsui, Ryo Sakasai, Masako Abe, Yusuke Kimura, Shoki Kajita, Wakana Torii, Yoko Katsuki, Masamichi Ishiai, Kuniyoshi Iwabuchi, Minoru Takata, Ryotaro Nishi

**Affiliations:** 1 Department of Biomedical Sciences, Ritsumeikan University, Kusatsu, Shiga 525-8577 Japan; 2 Department of Biochemistry I, Kanazawa Medical University, Kahoku, Ishikawa 920-0293 Japan; 3 Department of Late Effects Studies, Radiation Biology Centre, Graduate School of Biostudies, Kyoto University, Kyoto, Kyoto, 606-8501 Japan; 4 Central Radioisotope Division, National Cancer Centre Research Institute, Chuoku, Tokyo 104-0045 Japan; 5 School of Bioscience and Biotechnology, Tokyo University of Technology, Hachioji, Tokyo 192-0982 Japan

**Keywords:** Double-strand DNA breaks, Homologous recombination, Nuclear organization

## Abstract

The nucleus of mammalian cells is compartmentalized by nuclear bodies such as nuclear speckles, however, involvement of nuclear bodies, especially nuclear speckles, in DNA repair has not been actively investigated. Here, our focused screen for nuclear speckle factors involved in homologous recombination (HR), which is a faithful DNA double-strand break (DSB) repair mechanism, identified transcription-related nuclear speckle factors as potential HR regulators. Among the top hits, we provide evidence showing that USP42, which is a hitherto unidentified nuclear speckles protein, promotes HR by facilitating BRCA1 recruitment to DSB sites and DNA-end resection. We further showed that USP42 localization to nuclear speckles is required for efficient HR. Furthermore, we established that USP42 interacts with DHX9, which possesses DNA–RNA helicase activity, and is required for efficient resolution of DSB-induced R-loop. In conclusion, our data propose a model in which USP42 facilitates BRCA1 loading to DSB sites, resolution of DSB-induced R-loop and preferential DSB repair by HR, indicating the importance of nuclear speckle-mediated regulation of DSB repair.

## Introduction

Genomic DNA of mammalian cells does not distribute uniformly in the nucleus. The nucleus of mammalian cells contains various nuclear bodies, such as promyelocytic leukaemia bodies, Cajal bodies, speckles, paraspeckles, and nuclear speckles, which are membraneless structures that highly compartmentalize the nucleus^1,2^. Although these nuclear bodies play important roles in expressing genomic functions, including stress responses, messenger RNA (mRNA) splicing, and transcription^3^, the involvement of these nuclear bodies in the regulation of DNA repair remains elusive. Nuclear speckles (also designated interchromatin granule clusters) are self-organizing membraneless nuclear bodies that are detected as 20–50 irregularly shaped dots and localized in spaces between chromatins^4^. In situ hybridization and immunofluorescence staining of a representative nuclear speckle factor, SC35, revealed that nuclear speckles frequently localized next to transcriptionally active gene loci^5–9^. In accordance with their subnuclear localization, nuclear speckles are suggested to function as storage sites of mRNA-splicing factors and transcription factors^10–12^. In addition, some groups have suggested a direct contribution of nuclear speckles to splicing and transcriptional regulation^11,13–15^. Furthermore, in addition to poly(A)^+^ RNAs and non-coding RNAs^16,17^, mass spectrometry analysis of purified mouse liver nuclear speckles indicated that nuclear speckles are composed of not only proteins involved in RNA metabolism but also factors contributing to other cellular functions, such as apoptosis^18,19^, suggesting that nuclear speckles may play roles in a wide variety of cellular functions.

DNA double-strand breaks (DSBs) are likely the most cytotoxic DNA insults generated by endogenous sources and exogenous reagents such as ionizing radiation (IR). It is well known that dysfunctions in the DSB repair machinery cause human hereditary diseases, which often feature predisposition to tumorigenesis, immune deficiencies, and cellular hypersensitivity to IR, highlighting the importance of DSB repair for maintaining individual wellness and genome integrity^20,21^. DSBs are repaired mainly by two mutually exclusive pathways: non-homologous end-joining (NHEJ) and homologous recombination (HR). In contrast to error-permissive NHEJ, HR is thought to be an error-free pathway, since HR copies the DNA sequence from the undamaged sister chromatid in most cases^22,23^. It has been reported that DSBs generated in actively transcribed genes tend to be repaired by HR^24,25^, suggesting that HR could be preferentially chosen to accurately preserve genetic information for coding regions. In this study, we investigated the potential regulatory mechanism of HR mediated by nuclear speckles, which are also functionally associated with transcription.

## Materials and methods

### Cell lines and cell culture

All cell lines were cultured at 37 °C in a humidified 5% CO_2_ atmosphere. U2OS cells were cultured with Dulbecco’s modified Eagle’s medium (DMEM, Nacalai tesque, Kyoto, Japan) containing 10% foetal bovine serum (FBS, SIGMA-Aldrich, St. Louis, MO, USA), 100 U/ml penicillin (Nacalai tesque), 100 μg/ml streptomycin (Nacalai tesque), and 584 μg/ml l-glutamine. The U2OS USP42 KO cell line and its complemented cell lines stably expressing either GFP or GFP-USP42 (FL, ΔC or Δ946–1196) were cultured with identical media with 1 μg/ml puromycin (InvivoGen, San Diego, CA, USA) or with 1 μg/ml puromycin (InvivoGen) and 500 μg/ml Geneticin (ThermoFisher Scientific, Waltham, MA, USA), respectively.

### Transfection of plasmids and siRNAs

Cells were transfected with the plasmids or siRNAs (40 nM at final concentration) by Mirus TransIT-LT1 (Mirus Bio LLC, Madison, WI, USA) or HiperFect (Qiagen, Dusseldorf, Germany) according to the manufacturer’s instructions, respectively. The plasmid coding siRNA-resistant GFP-fused DHX9 was constructed by introducing mutations into wild-type DHX9 with the primers: (1) TAAACGAGCGATGCTGAACATGATCCGTCAGA and (2) GCATGCGCTCGTTTACGGGGTCCAACTGGCTGA. See Table S1 for the siRNAs used in this study.

### Direct-Repeat GFP (DR-GFP) assay

The DR-GFP assay was carried out as previously described^26^. HR repair efficiency was investigated by a chromosomal DSB-induced gene conversion assay system with transient expression of the I-SceI restriction enzyme in U2OS cells carrying a DR-GFP reporter as previously described^27^.

### Cell extract preparation and immunoblotting analysis

Except for sample preparation for mass spectrometry analysis and immunoprecipitation, cell extracts were prepared with CSK buffer [10 mM PIPES (pH 6.8), 3 mM MgCl_2_, 1 mM ethylene glycol tetraacetic acid (EGTA), 0.1% Triton X-100 and 300 mM sucrose] containing 300 mM NaCl, 1 x Protease Inhibitor cocktail ethylenediaminetetraacetic acid (EDTA)-free (PI, Roche, Basel, Switzerland), 10 mM NaF (Nacalai tesque), 20 mM N-ethylmaleimide (NEM, Nacalai tesque), and 0.25 mM phenylmethylsulfonyl fluoride (PMSF, SIGMA-Aldrich). The cells were washed twice with ice-cold PBS and incubated with an appropriate volume of CSK buffer for 1 h on ice with occasional mixing. Soluble fractions were collected by centrifugation at 20,000 × *g* for 10 min at 4 °C. Residual chromatin fractions (pellet fractions) were washed twice with identical buffer and then solubilized by sonication (UD-100, 40% output, 30 s, TOMY, Tokyo, Japan). Where indicated, cells were incubated with 2.5 μg/ml tubercidin (Sigma-Aldrich) for 2 h and/or 1 μM CPT for 1 h. For mass spectrometry analysis and immunoprecipitation, cells were washed twice with ice-cold PBS and collected with an appropriate volume of ice-cold PBS, followed by centrifugation at 10,000 × *g*, for 1 min. Cells were lysed with IP lysis buffer [20 mM Tris–HCl (pH 7.5), 2 mM MgCl_2_, 0.5% NP-40, and 10% glycerol] containing 40 mM NaCl, 1 × PI, 10 mM NaF, 20 mM NEM, 0.25 mM PMSF, and 50 U/ml Benzonase (Merck Millipore, Burlington, MA, USA) and incubated at room temperature for 5 min. Soluble fractions were prepared by rotation at 4 °C for 1 h after adjusting the NaCl concentrations to 300 mM, followed by centrifugation at 20,000 × *g* for 10 min at 4 °C. When cell extract was prepared by mechanical shearing, cells suspended with CSK buffer containing 150 mM NaCl, 1 × PI, 10 mM NaF, 20 mM NEM, and 0.25 mM PMSF were lysed by passing through 23 G needle 10 times. After incubating at 4 °C for 1 h, soluble fraction was obtained by centrifugation at 20,000 × *g*, for 10 min at 4 °C. The protein concentrations of cell extracts were determined with Coomassie Protein Assay Reagent (Thermo Fisher Scientific) with bovine serum albumin (BSA) standard (TAKARA BIO, Shiga, Japan). The antibodies used in this research are described in Table S2. All immunoblotting data was replicated at least twice in the laboratory.

### Immunofluorescence staining

For subcellular localization analysis of USP42, cells were fixed with 4% paraformaldehyde (PFA) for 15 min at room temperature and then permeabilized by incubation with 0.2% Triton X-100 in PBS for 5 min at room temperature. To examine 53BP1 foci formation, cells that were irradiated with 2 Gy of IR (Faxitron RX-650, Tucson, AZ, USA) and then incubated for 15 min were pre-extracted prior to fixation with pre-extraction buffer [10 mM Pipes (pH 6.8), 3 mM MgCl_2_, 3 mM EDTA, 0.5% Triton X-100, 0.3 M sucrose, and 50 mM NaCl] for 5 min on ice. For the purpose of investigating RAD51 and BRCA1 foci formation, cells that were irradiated with 2 Gy of IR and then incubated for 6 h were pre-extracted with 0.2% Triton X-100 for 1 or 5 min, respectively, and then fixed with 3% PFA and 2% sucrose in PBS for 15 min. Hereafter, the samples were washed twice with 0.1% Tween 20 in PBS after each procedure. After incubating cells with blocking buffer A [5% FBS, 0.1% Triton X-100 in PBS] for 30 min, the cells were sequentially incubated with primary antibodies for 1 h and with secondary antibodies for 30 min diluted in blocking buffer A for USP42 localization and 53BP1 foci formation analysis. For RAD51 and BRCA1 foci formation analysis, blocking buffer B (2% BSA in PBS) was used instead and incubated for 1 h prior to the incubation with the antibodies. For detecting pRPA2 S4/S8 foci, cells were fixed with 3% PFA–2% sucrose in PBS for 15 min and then permeabilized with 0.2% TritonX-100 in PBS for 5 min at room temperature. Subsequently, cells that were blocked with Blocking One (Nacalai tesque) for 20 min at room temperature were incubated with an anti-pRPA2 S4/S8 antibody for 1 h and then with secondary antibody for another 1 h. Following nuclei staining with 1 μg/ml of 4′,6-diamidino-2-phenylindole (DAPI) solution for 10 min, the samples were sealed with VECTASHIELD (VECTOR LABORATORIES, Burlingame, CA, USA), and images were taken with a confocal microscope (TCS SP5, Leica, Wetzlar, Germany) or BZ-9000 (KEYENCE, Osaka, Japan) and analysed with a software (LAS AF, Leica). To analyse 53BP1 foci formation, images were taken by IN Cell Analyzer 2000 (GE Healthcare, Chicago, IL, USA), and then cells were classified into the S, G2, and G1 phases based on the signal intensity of anti-CENPF antibody staining with software (IN Cell Investigator, GE Healthcare).

### Cell cycle profile analysis

The cell cycle profile was analysed with BrdU incorporation as previously described^26^.

### Quantitative DNA-end resection assay

The efficiency of DNA-end resection was measured in a quantitative manner, as previously described^26^. Briefly, cells were labelled with 30 μM of BrdU for 24 h prior to 1 μM CPT treatment for 1 h. The cells were processed for staining with an anti-BrdU antibody under non-denaturing conditions, followed by incubation with appropriate secondary antibodies, and then analysed with LSRFortessa (BD Biosciences, San Jose, CA, USA). The signal intensity of the anti-BrdU antibody in S-phase cells detected with propidium iodide staining was obtained by FlowJo software (Becton Dickinson, Franklin Lakes, NJ, USA). After subtraction of the CPT non-treated background signal, the mean intensity of the anti-BrdU antibody staining of each sample was normalized to that seen immediately after CPT treatment with siRNA control.

### Establishment of the USP42 KO cell line and its complemented cell lines

The gRNA sequence (5′-ATTGGTTTAATAACGTCCCC-3′) was cloned between the BamHI site and the BsmBI site of the pCas-Guide vector (OriGene, Rockville, MD, USA). Sequences of the homology arms flanking the PAM site were cloned into pDonor-D09 (GeneCopoeia, Rockville, MD, USA). U2OS cells co-transfected with these plasmids were cultured for 14 days with puromycin and screened for loss of USP42 expression by immunoblotting. To obtain complemented cell lines, the USP42 KO cell line was transfected with the plasmids encoding GFP or GFP-USP42 (FL, ΔC or Δ946–1196) and cultured for 14 days with 1 mg/ml Geneticin.

### Clonogenic cell survival assay

Clonogenic viability was examined using a colony formation assay. Briefly, 48 h after the initial transfection with siRNAs, cells were seeded in six-well plates and treated with acute IR on the following day. For the assay with the USP42 KO cell line, cells were seeded in six-well plates one day before irradiation. Colonies were stained with crystal violet solution [2% crystal violet (Sigma-Aldrich) in 10% ethanol] 10–13 days after IR treatment.

### Immunoprecipitation

Soluble fractions of cell extracts were immunoprecipitated with an anti-GFP antibody coupled to magnetic beads (GFP-Trap_MA, ChromoTek, Planegg-Martinsried, Germany) or an anti-HA antibody coupled to agarose beads (EZview Red Anti-HA Affinity Gel, SIGMA-Aldrich) by rotation overnight at 4 °C. The beads were washed six times with the buffer used for cell extract preparation and bound proteins were eluted by boiling at 95 °C for 10 min with 1 x Laemmli SDS buffer [62.5 mM Tris–HCl (pH 6.8), 2% sodium dodecyl sulphate, 10% glycerol and 0.02% bromophenol blue, 6.25% β-mercaptoethanol and 300 mM NaCl].

### Mass spectrometry analysis

Cell extracts were prepared with the Benzonase-based method (see above) from U2OS cells transiently transfected with pEGFP-C1-USP42 or pEGFP-C1 as a negative control. Immunoprecipitates generated with an anti-GFP antibody were washed six times with IP lysis buffer containing 300 mM NaCl, 1 x PI, 10 mM NaF, 20 mM NEM, and 0.25 mM PMSF and then collected by boiling at 95 °C for 10 min with 1 x Laemmli SDS buffer. Immunoprecipitated samples were digested in-solution and analysed using nanoliquid chromatography tandem mass spectrometry (nano-LC–MS/MS) provided by Filgen (Nagoya, Japan).

### Slot blot analysis for R-loop quantification

Slot blot analysis was basically performed as previously described^28^. Briefly, genomic DNA (1 μg) was transferred to a nylon membrane using slot blot apparatus in duplicate for R-loop detection with an S9.6 antibody and DNA staining with ethidium bromide. The signal intensity obtained with an S9.6 antibody was normalized by the signal intensity with ethidium bromide.

### Statistical analysis

The data were analysed by quantile–quantile plot to test whether these follow normal distribution. All statistical analyses were performed using a standard two-sided Student’s *t*-test (equal variance). In all experiments, sample size (*n*) indicates biological replicate. For immunoblotting and immunofluorescent analysis, the experiments were repeated at least twice. For experiments subjected to statistical analysis, exact numbers of biological replicates were provided in figure legends.

## Results

### A focused screen identified transcription-related nuclear speckle factors as HR regulators

**Fig. 1:**
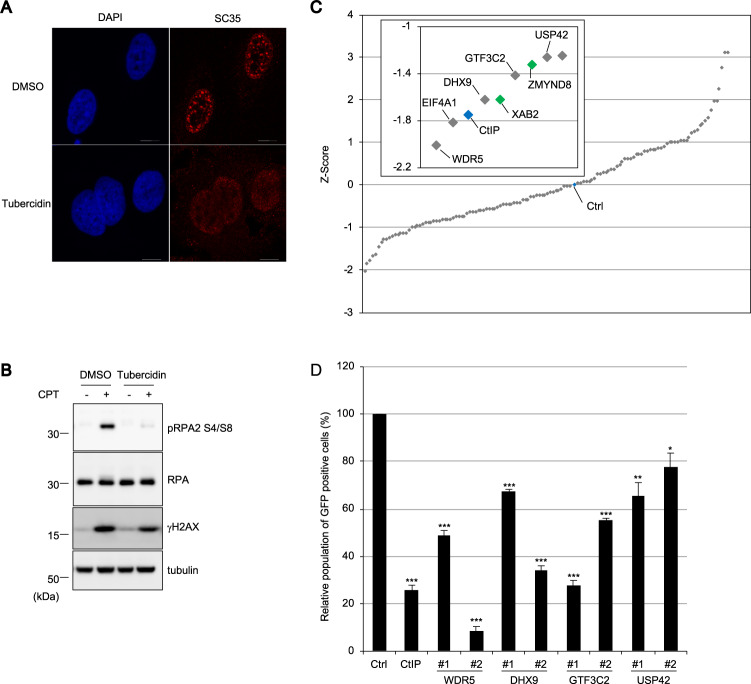
A focused screen identified transcription-related nuclear speckle factors as HR regulators. **a** U2OS cells treated with tubercidin or mock treated (dimethyl sulfoxide: DMSO) were subjected to immunofluorescence staining with the anti-SC35 antibody. Scale bar: 10 μm. **b** U2OS cells were incubated with tubercidin or DMSO followed by treatment with CPT. Cell extracts were subjected to immunoblotting analysis with the indicated antibodies. **c** Screen for nuclear speckle factors involved in HR regulation. The DR-GFP assay was performed with siRNA pools targeting nuclear speckle factors. The siRNA targeting CtIP or luciferase (control: Ctrl) was used as a positive or negative control, respectively, and indicated with blue markers. Homology directed repair efficiency is plotted as a *Z*-score. The inset magnifies the data for top hits with gene symbols. The genes previously reported to be involved in HR regulation are indicated with green. **d** A DR-GFP assay was carried out with two independent siRNAs targeting selected top hits from the initial screen. GFP-positive cell populations were normalized to mock treatment (Ctrl), which was set to 100% (mean ± SEM, *n* = 3). *p < 0.05. **p < 0.01. ***p < 0.005.

To investigate whether nuclear speckles spatially contribute to proper DSB responses, especially HR, camptothecin (CPT)-induced phosphorylation of RPA2 on Ser4 and Ser8 (pRPA2 S4/S8), which is thought to be an indicative of the early HR process, was examined in the presence of tubercidin, which induces the dispersion of some nuclear speckle factors, including SRSF1, SC35, and poly(A)^+^ RNA (Fig. 1a)^29^. As shown in Fig. 1b, tubercidin treatment resulted in reduced phosphorylation of RPA2 after CPT treatment, suggesting that the integrity of nuclear speckles or the localization of some nuclear speckle factors to distinct foci is required for efficient HR. To evaluate the effect of nuclear speckle factors on HR, we investigated the frequency of non-crossover gene conversion-mediated HR with a DR-GFP assay by knocking down each nuclear speckle factors. A short interfering RNA (siRNA) library comprised 128 siRNAs targeting potential nuclear speckle factors identified by proteomics analysis (Table S1), while proteins involved in mRNA splicing were excluded because of a preciously suggested intervention for DSB repair^30^. The siRNA library also included siRNAs targeting the deubiquitylating enzyme USP42, which showed a dot-like localization similar to nuclear speckles^26,31^ and was found actually to colocalize with SC35 and to disperse in the nucleoplasm to a lesser extent (Fig. S1A). In this screening, two siRNAs were pooled for each target. The efficiencies of HR were *Z*-score normalized, identifying several factors that are WDR5, EIF4A, DHX9, GTF3C2, and USP42 as potential novel HR regulators (Fig. 1c). In addition to a positive control (siCtIP), our screen also identified XAB2 and ZMYND8 that had already been suggested to promote HR, suggesting robustness of the screen^32,33^. Furthermore, the DR-GFP assay was carried out with two individual siRNAs targeting WDR5, DHX9, GTF3C2, and USP42, verifying the results of the initial screen (Figs. 1d and S1B). Since USP42 is a hitherto unidentified nuclear speckle factor and is a unique deubiquitylating enzyme that localizes to nuclear speckles, we decided to focus on USP42 to reveal a nuclear speckle-mediated HR regulation.

### USP42 promotes HR by facilitating DNA-end resection

**Fig. 2:**
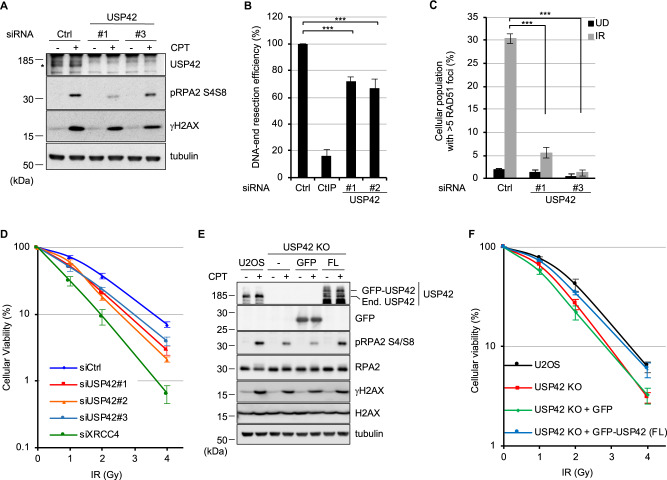
USP42 promotes HR by facilitating DNA-end resection. **a** U2OS cells transfected either with siRNA control (Ctrl) or siRNA targeting USP42 were treated with CPT or mock treated. Cell extracts were analysed by immunoblotting with the indicated antibodies. Asterisk indicates endogenous USP42. Immunoblotting with the anti-γH2AX antibody was used as a control for DNA damage induction. **b** A DNA-end resection assay was carried out with the cells transfected with the indicated siRNAs (mean ± SEM, *n* = 3). The siRNA targeting CtIP was a positive control. **c** RAD51 foci formation efficiency was examined with cells transfected with the indicated siRNAs (mean ± SEM, *n* = 3). The population of cells with >5 RAD51 foci was plotted. **d** Cells transfected with the indicated siRNAs were subjected to a clonogenic survival assay (mean ± SEM, *n* = 4 for siCtrl and siUSP42#2, *n* = 3 for siUSP42#1, siUSP42#3, and siXRCC4). The siRNA targeting XRCC4 was used as a positive control. **e** U2OS, USP42 KO, or USP42 KO cells complemented with either GFP or GFP-USP42 (FL) were treated with CPT and subjected to immunoblotting analysis with the indicated antibodies. End. USP42: endogenous USP42. **f** The indicated cell lines were subjected to a clonogenic survival assay [mean ± SEM, *n* = 6 for U2OS, USP42 KO + GFP and USP42 KO + GFP-USP42 (FL), *n* = 5 for USP42 KO]. ***p < 0.005.

To understand how USP42 promotes HR, the effects of USP42 depletion with siRNAs on various aspects of HR were investigated. Depletion of USP42 with two individual siRNAs resulted in reduced phosphorylation of RPA2 (pRPA2 S4/S8) upon CPT treatment, suggesting that USP42 could function in early phases of HR (Fig. 2a). Thus, we examined the efficiency of DNA-end resection that generates 3′ single-stranded DNA overhangs by nucleolytic degradation of the 5′ terminated stand of the DSB upon CPT treatment. For this purpose, cells that were labelled with BrdU and then treated with CPT were processed for immunostaining with an anti-BrdU antibody under non-denaturing condition, specifically detecting BrdU in single-strand DNA region generated upon CPT treatment but not in double-stand DNA. The signal intensity with an anti-BrdU antibody staining that corresponds to the length of generated single-strand DNA was measured by FACS analysis. The result indicated that USP42 is required for efficient DNA-end resection (Fig. 2b). Since DNA-end resection also occurs during single-strand annealing (SSA) and break-induced replication (BIR), which are RAD51-independent DSB repair pathways, RAD51 foci formation was tested. Depleting USP42 resulted in significantly reduced RAD51 foci formation, suggesting that USP42 functions upstream of RAD51 recruitment to DSB sites and promotes HR. (Figs. 2c and S2A). In addition, USP42 might also regulate RAD51-independent DSB repair pathways including SSA and BIR by promoting DNA-end resection. These results were not due to smaller population in S and G2 phase (Figs. S2B and S2C). Furthermore, USP42 depletion with three independent siRNAs sensitized cells to IR, indicating its physiological importance (Fig. 2d). In addition, USP42 depletion did not affect the protein levels of HR factors involved in DNA-end resection, such as the MRE11–RAD50–NBS1 complex and CtIP (Fig. S2D). Furthermore, to carry out rescue experiments, we created a USP42 knockout U2OS cell line (USP42 KO). This cell line recapitulated the phenotypes caused by USP42 knockdown, which were rescued by stable expression of exogenous GFP-tagged USP42 but not of GFP (Figs. 2e, f and S2E, S2F). Altogether, these results indicated that USP42 promoted HR and cellular viability by facilitating DNA-end resection.

### USP42 is required for efficient recruitment of BRCA1 to DSB sites

**Fig. 3:**
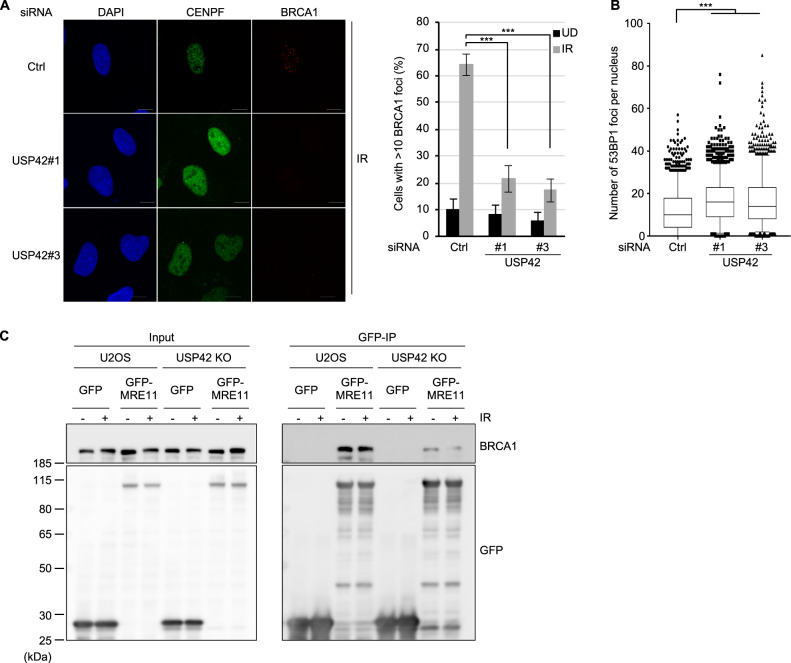
USP42 is required for efficient recruitment of BRCA1 to DSB sites. **a** BRCA1 foci formation efficiency was examined with cells transfected with the indicated siRNAs. (Left) The representative images are shown. (Right) The population of cells with >10 BRCA1 foci was plotted (mean ± SEM, *n* = 3). The cells positive for CENPF staining were analysed. See Fig. S3 for the representative images of undamaged condition. **b** 53BP1 foci formation efficiency was examined with cells transfected with the indicated siRNAs. The number of foci per nucleus after IR were plotted as a Box and Whiskers plot (median, 5–95 percentile, *n* = 3). The cells positive for CENPF staining were analysed. **c** U2OS or USP42 KO cells were transfected with plasmids encoding either GFP or GFP-MRE11. Cell extracts were subjected to immunoprecipitation with an anti-GFP antibody followed by immunoblotting analysis with the indicated antibodies. ***p < 0.005.

As DSBs generated in transcriptionally active regions tend to be repaired by HR, we speculated that USP42, which localizes close to actively transcribed loci, may favour HR over NHEJ for these DSBs. Since BRCA1 and 53BP1 had been known to be key regulatory factors for DSB repair pathway choice^34^, recruitment of these proteins to DSB sites were investigated. Supporting our idea, knockdown of USP42 resulted in reduced BRCA1 foci formation, which promotes HR, in CENPF-positive cells, whereas the number of 53BP1 foci per cell, which counteracts BRCA1 loading and HR, was increased (Figs. 3a, b and S3). Furthermore, we investigated the effect of USP42 on the interaction between BRCA1 and MRN complex by immunoprecipitation with GFP-fused MRE11. As shown in Fig. 3c, although the interaction between MRE11 and BRCA1 was readily detected in U2OS cells, this interaction was attenuated in USP42 KO cells. These results suggest that USP42 facilitates BRCA1 recruitment to DSB sites by mediating the interaction between BRCA1 and MRN complex.

### Nuclear speckle localization of USP42 is required for efficient HR

**Fig. 4:**
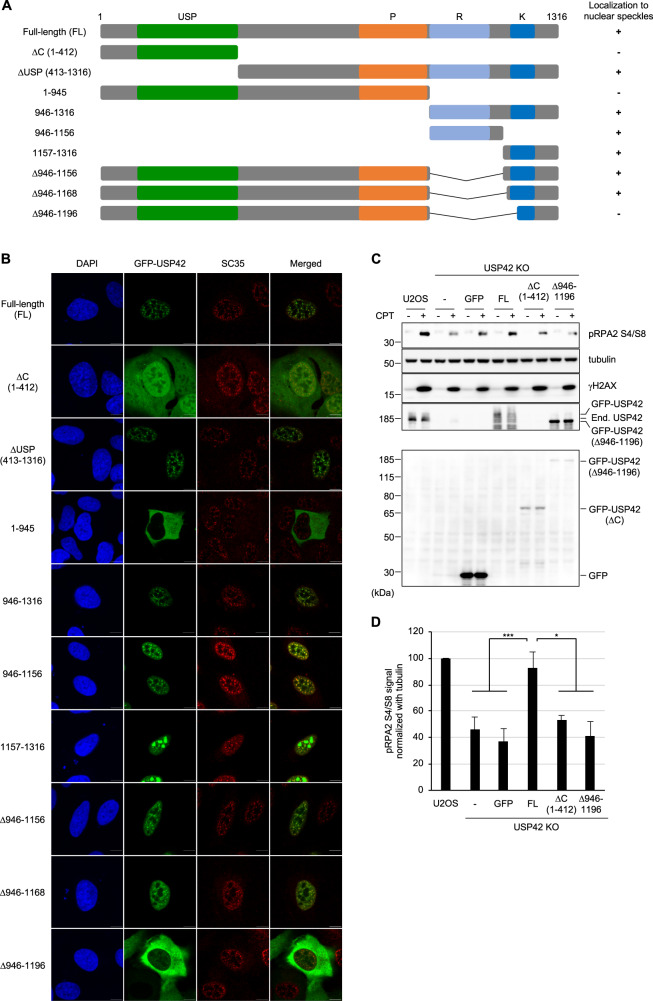
Nuclear speckle localization of USP42 is required for efficient HR. **a** Schematic representation of USP42 protein and truncated mutants. The numbers represent amino acid residues. USP: ubiquitin-specific protease domain, P: proline-rich region, R: arginine-rich region, K: lysine-rich region. Localization to nuclear speckles of the indicated mutants are shown on the right. **b** Subcellular localization of various GFP-fused USP42 proteins. Cells transfected with the plasmids encoding the indicated GFP-USP42 were subjected to immunofluorescence staining with an anti-SC35 antibody. Scale bar: 10 μm. **c** U2OS or USP42 KO cells complemented with either GFP or GFP-USP42 (FL, ΔC or Δ946–1196) were treated with CPT and subjected to immunoblotting analysis with the indicated antibodies. Note that endogenous USP42 (End. USP42) and GFP-USP42 (FL) could only be detected in pellet fraction with an anti-USP42 antibody, while GFP and GFP-USP42 (ΔC) were detected in soluble fraction with an anti-GFP antibody. GFP-USP42 (Δ946–1196) was detected in both fractions. **d** The signal intensities of phosphorylated RPA2 that were normalized to the signal intensity of tubulin were plotted [mean ± SEM, *n* = 11 for U2OS and USP42 KO, *n* = 10 for USP42 KO + GFP, *n* = 8 for USP42 KO + GFP-USP42 (FL), *n* = 5 for USP42 KO + GFP-USP42 (ΔC), *n* = 3 for USP42 KO + GFP-USP42 (Δ946–1196)]. *p < 0.05. ***p < 0.005.

In ensuing studies, we examined whether the subnuclear localization of USP42 plays a role in its function in HR by elucidating the nature of the nuclear speckle localization of USP42. As shown in Fig. 4a, except for the ubiquitin-specific protease (USP) domain that is a deubiquitylating domain, USP42 does not contain obvious functional domains, whereas proline (P)-rich, arginine (R)-rich and lysine (K)-rich regions are found in its carboxyl-terminal half. First, we asked whether the enzymatic activity of USP42 is required for its nuclear speckle localization (Fig. 4a, b). While the USP42 mutant lacking the USP domain (ΔUSP) colocalized with SC35, the ΔC mutant (1–412 amino acids (a.a.) residues) that included the USP domain showed dispersed nuclear and partial cytoplasmic localization, suggesting that the enzymatic activity of USP42 is dispensable for its nuclear speckle localization. To identify the domain(s) responsible for nuclear speckle localization, the localization of various truncated mutants of USP42 was examined (Fig. 4a, b). USP42 lacking the C-terminal region (1–945 a.a.) almost completely localized to the cytoplasm, whereas the C-terminal region (946–1316 a.a.) was sufficient for nuclear and nuclear speckle localization, indicating nuclear and nuclear speckle localization signal domain reside within C-terminal region. Further truncation of this C-terminal region identified two segments, 946–1156 a. a. and 1157–1316 a.a. that are sufficient for nuclear speckle localization of USP42, although the former region represented clearer colocalization with SC35. Further fragmentation of the former region resulted in a diffused and large dot-like pattern in the nucleus that were not likely to be nuclear speckles (Figs. S4A and S4B), suggesting that the integrity of this region (946–1156 a.a.) is important for nuclear speckle localization of USP42. Deleting these regions from the USP42 protein revealed that a domain from 946 to 1196 residues is required for nuclear speckle localization of USP42. Amino acid sequence of this region (946–1196 a.a.) weakly matched the arginine/serine (RS) repeat motif, which was previously reported as a nuclear speckle localization signal (Fig. S4C)^35–37^. Since it is suggested that the intrinsically disordered region (also known as the low complexity domain) plays a pivotal role in the formation of membraneless organelles by liquid–liquid phase separation (LLPS)^38^, USP42 was analysed for an intrinsically disordered region (Fig. S4D)^39^. As expected, USP42 was predicted to be mostly disordered, including a nuclear speckle localization signal domain (946–1196 a.a.). Finally, we examined CPT-induced signalling in USP42 KO cell lines that stably express either GFP or GFP-fused USP42 (full-length: FL, ΔC or Δ946–1196 a.a.) (Fig. 4c, d). Although exogenously expressed GFP-USP42 (FL) restored CPT-induced phosphorylation of RPA2, cells expressing truncated mutants of USP42 that lost nuclear speckle localization failed to rescue this phenotype, suggesting that nuclear speckle localization of USP42 is indispensable for proper HR progression.

### USP42 is epistatic with DHX9 in the cellular survival after DSB induction and promotes resolution of DSB-induced R-loop

**Fig. 5:**
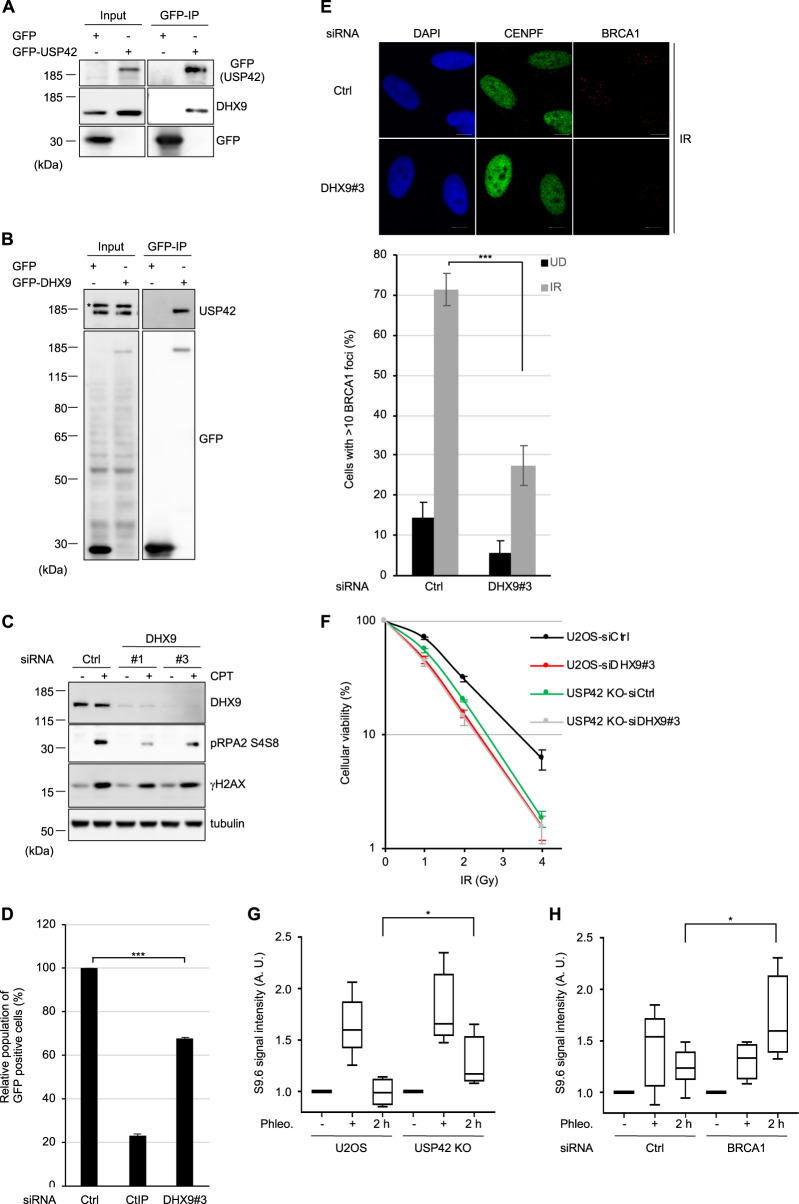
USP42 is epistatic with DHX9 in the cellular survival after DSB induction and promotes resolution of DSB-induced R-loop. **a**, **b** The interaction between USP42 and DHX9 was tested by coimmunoprecipitation with either GFP-USP42 **a** or GFP-DHX9 **b** followed by immunoblotting analysis with the indicated antibodies. Transfection with a plasmid encoding GFP was a negative control. **c** U2OS cells transfected with the indicated siRNAs were treated with CPT and then subjected to immunoblotting analysis with the indicated antibodies. **d** A DR-GFP assay was performed with the cells transfected with the indicated siRNAs (mean ± SEM, *n* = 3). **e** BRCA1 foci formation efficiency was examined with cells transfected with the indicated siRNAs. (Upper) The representative images are shown. (Lower) The population of cells with >10 BRCA1 foci was plotted (mean ± SEM, *n* = 3). The cells positive for CENPF staining were analysed. See Fig. S4B for the representative images of undamaged condition. **f** U2OS or USP42 KO cells transfected with the indicated siRNAs were subjected to a clonogenic survival assay (mean ± SEM, *n* = 5). **g**, **h** The indicated cells **g** or cells transfected with the indicated siRNAs **h** were treated with phleomycin (Phleo., +) and then further cultured for 2 h upon removal of phleomycin (2 h). Purified genomic DNA was subjected to slot blot analysis with an S9.6 antibody. Signal intensity was normalized to mock-treated samples (−), and then plotted as a Box and Whiskers plot (median, 5–95 percentile, *n* = 5 for U2OS, USP42 KO, and siBRCA1, *n* = 7 for siCtrl). *p < 0.05, ***p < 0.005.

In order to investigate how USP42 fulfils its function in HR, mass spectrometry analysis of affinity-purified GFP-USP42-interacting proteins was performed with a negative control (i.e., GFP). The mass spectrometry analysis identified 161 proteins that specifically interact with USP42 (Table S3). Recently, regulation of HR mediated by DNA–RNA hybrid structure (R-loop) was reported^40^. In addition, γH2AX (Ser139-phosphorylated H2AX) and RNA polymerase II was shown to interact with DHX9 (also known as RHA or NDHII) that belongs to DEAH RNA helicase family and has activity to resolve R-loop^41–43^. Because of these knowledges, in ensuing study we focused on DHX9 that is one of the top hits of our mass spectrometry analysis. Firstly, we confirmed that USP42 interacted with DHX9 in a reciprocal manner by coimmunoprecipitation (Fig. 5a, b). It is worth noting that the interaction between USP42 and DHX9 was independent of RNA and DNA, since immunoprecipitation was carried out in the presence of Benzonase (also see section “Materials and methods”). To investigate whether DHX9 is involved in HR, we assessed CPT-induced phosphorylation of RPA2 with two independent siRNAs targeting DHX9. Depletion of DHX9 resulted in a reduced pRPA2 S4/S8 signal (Fig. 5c). Consistently, pRPA2 S4/S8 signal induced by CPT treatment was disturbed by depleting endogenous DHX9 (data not shown), which tended to be rescued by transient over-expression of siRNA-resistant GFP-DHX9 (Fig. S5A), suggesting that the phenotype caused by siRNA targeting DHX9 could not be an off-target effect. In addition, depleting DHX9 resulted in impaired HR activity with the DR-GFP assay (Figs. 1d and 5d) without affecting the cell cycle profile (Fig. S5B). Furthermore, DHX9 depletion resulted in reduced BRCA1 foci formation (Figs. 5e and S5C). We found that depletion of DHX9 did not result in obvious collapse of nuclear speckles (Fig. S5D). These findings prompted us to examine whether USP42 and DHX9 function in the same axis in HR. While depletion of DHX9 in U2OS cells resulted in increased sensitivity to IR compared to control siRNA, knocking down DHX9 did not confer further sensitivity to IR in USP42 KO cells (Fig. 5f), indicating that USP42 is epistatic with DHX9, and both proteins are required for proper HR. We have also performed a DR-GFP assay to investigate whether DHX9 over-expression can rescue the HR defect caused by USP42 depletion (Fig. S5E). HR efficiency that was reduced by knocking down endogenous USP42 was not significantly reverted by transient over-expression of mCherry-fused DHX9, although a trend was seen in that HR efficiency was increased. This may suggest that USP42 is required for DHX9 to fully function in HR. Furthermore, since it was previously suggested that DHX9 promotes R-loop resolution in vivo and in vitro^41,44,45^, we speculated that USP42 could facilitate R-loop resolution together with DHX9 in HR. In line with this idea, the signal intensity of the S9.6 antibody that specifically detects the R-loop was increased upon DSB induction and remained increased in USP42 KO cells compared to parental U2OS cells (Fig. 5g), indicating that USP42 is required for DSB-induced R-loop resolution. We also found that DSB-induced R-loop persisted longer in BRCA1-depleted cells in comparison to control cells, suggesting that BRCA1 is required for the efficient R-loop resolution (Figs. 5h and S5F). Finally, we examined physical interaction between BRCA1 and DHX9 by immunoprecipitation (Fig. S5G). While we did not detect the interaction between BRCA1 and DHX9 when cell extract was prepared with Benzonase degrading both DNA and RNA, the interaction between these proteins was observed if cell extract was prepared by mechanical shearing (also see section “Materials and methods”), indicating that these proteins interacted indirectly in a manner mediated with DNA or RNA. Based on these results, we propose a model in which USP42 and DHX9 promote BRCA1 loading to DSB sites near nuclear speckles, which facilitates DSB-induced R-loop resolution and preferential DSB repair by HR.

## Discussion

In this study, we identified several nuclear speckle factors potentially involved in HR regulation based on a DR-GFP reporter assay. Among these were transcription regulators, including WDR5, ZMYND8, and USP42. It is well known that WDR5 forms the histone H3 lysine 4 methyltransferase complex with mixed lineage leukaemia and promotes RNA polymerase II-dependent transcription activation^46^. ZMYND8 was reported to play roles in activating transcription^47^. USP42 was suggested to enhance transcription by deubiquitylating histone H2B^31^. These findings might suggest that active transcription in the vicinity of nuclear speckles is required for efficient HR or favouring HR over NHEJ, consistent with the idea termed transcription-associated HR^40^. Supporting this idea, it was recently suggested that WDR5, BRCA1, and BARD1 were functionally connected to suppress DNA damage during reprogramming^48^. ZMYND8 also promotes HR via multivalent binding to chromatin, including histone H4 acetylations^32,49^. To precisely understand the spaciotemporal regulation of HR, it will be essential to specifically examine repair of DSBs located next to nuclear speckles. It is worth noting that GTF3C2, which is a subunit of transcription factor TFIIIC and plays roles in the expression of non-coding RNAs, such as human Alu RNA and mouse B2 RNA by RNA polymerase III, was identified in our screen. These non-coding RNAs were upregulated by stresses such as etoposide and IR^50^, and inhibited transcription by RNA polymerase II^51,52^. Therefore, it will be an exciting research area to explore a cross-talk between non-coding RNA-mediated transcription inhibition and DSB repair regulation mediated by nuclear speckles.

By focusing on USP42, we indicate that USP42 promotes HR by aiding interaction between BRCA1 and MRN complex, recruiting BRCA1 to DSB sites, facilitating resolution of the DSB-induced R-loop and promoting DNA-end resection. In addition to the function of BRCA1 preventing R-loop accumulation under physiological conditions^53,54^, our study indicated that BRCA1 also contributes to resolve R-loop in the context of DSB repair in a manner dependent on USP42. On the other hand, consistent with the previous report^55^, we found that depleting BRCA1 resulted in quite marginal DNA-end resection defects, and no obvious reduction of pRPA2S4/S8 after CPT treatment (Figs. S5F and S5H). Because USP42 depletion resulted in clear reduction of phosphorylated RPA2 and DNA-end resection, these may suggest that USP42 has another function in promoting DNA-end resection in addition to BRCA1 loading to DSB sites. Since it is evident that processing of the R-loop could positively and negatively influence DSB repair^56^, how the R-loop affects DSB repair might be dependent on the stage of DSB repair, transcriptional activity, and the genomic location of DSB. Such complexity of R-loop-mediated regulation of DSB repair can explain why various proteins, including RAD52-XPG and SETX, have been implicated in R-loop resolution^40,54,57^.

Through domain mapping analysis of USP42, an intrinsically disordered region (946–1196 a.a.) was identified as a signal domain for nuclear speckle localization, which is important for HR promotion. Interestingly, DHX9 is also predicted to contain another type of disordered domain, the prion-like domain that is suggested to play roles in phase separation^58^. LLPS was previously implicated in transcriptional regulation by increasing local concentrations of proteins and compartmentalization^38^; thus, our data may suggest that DSB repair is also regulated by LLPS-dependent manner. It had also been suggested that LLPS was regulated by RNA binding to intrinsically disordered regions such as RS repeats^59^. Therefore, it will be interesting to examine whether LLPS mediated by RNA, including non-coding RNAs, contributes to DNA repair.

Our results suggested that regulation of HR mediated by nuclear speckles plays key roles for cellular viability after DSB induction, highlighting the importance of spatial regulation of HR. Thus, in order to understand the nuclear compartmentalization-mediated regulation of DSB repair, revealing driving force of nuclear body formation and chromosome dynamics will be essential.

## Supplementary Information

Supplementary Information
