# Supplementary material for: USP42 enhances homologous recombination repair by promoting R-loop resolution with a DNA–RNA helicase DHX9
Source: Oncogenesis. 2020 Jun 15;9(6):60. doi: 10.1038/s41389-020-00244-4 (PMC7296013; doi:10.1038/s41389-020-00244-4)
Supplement: Supplementary file 1 — Supplementary Information [file 41389_2020_244_MOESM1_ESM.pdf]

Supplementary Information for

USP42 enhances homologous recombination repair by promoting R-loop resolution with a DNA-RNA helicase DHX9

Misaki Matsui[1], Ryo Sakasai[2], Masako Abe[3], Yusuke Kimura[1], Shoki Kajita[1], Wakana Torii[1], Yoko Katsuki[3], Masamichi Ishiai[4], Kuniyoshi Iwabuchi[2], Minoru Takata[3] and Ryotaro Nishi[*,1,5]

[1]Department of Biomedical Sciences, Ritsumeikan University, Kusatsu, Shiga, 525-8577, Japan

[2]Department of Biochemistry I, Kanazawa Medical University, Kahoku, Ishikawa, 920-0293, Japan

[3]Department of Late Effects Studies, Radiation Biology Centre, Graduate School of Biostudies, Kyoto University, Kyoto, Kyoto, 606-8501, Japan

[4]Central Radioisotope Division, National Cancer Centre Research Institute, Chuoku, Tokyo, 104-0045, Japan

[5]School of Bioscience and Biotechnology, Tokyo University of Technology, Hachioji, Tokyo, 192-0982, Japan

[*]Correspondence to Ryotaro Nishi: nishirtr@stf.teu.ac.jp

This PDF file includes: Supplementary Figures S1-S5

Supplementary Table S1-S3

Figure S1

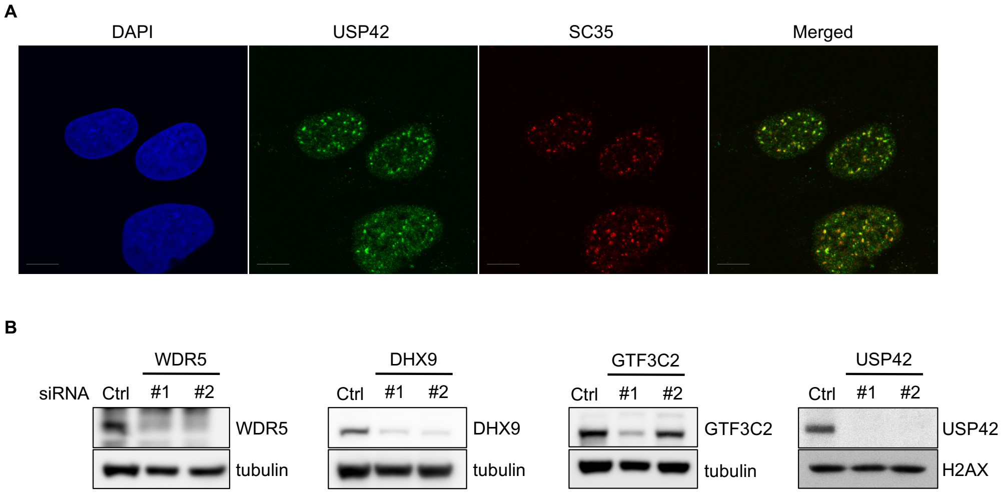

Supplementary Figure S1 A focused screen identified transcription-related nuclear speckle factors as HR regulators

(A) U2OS cells were subjected to immunofluorescence staining with anti-USP42 and anti-SC35 antibodies. Nuclei were stained with DAPI. Scale bar: 10 µm. (B) U2OS cells transfected with the indicated siRNAs were subjected to immunoblotting analysis with the indicated antibodies.

Figure S2

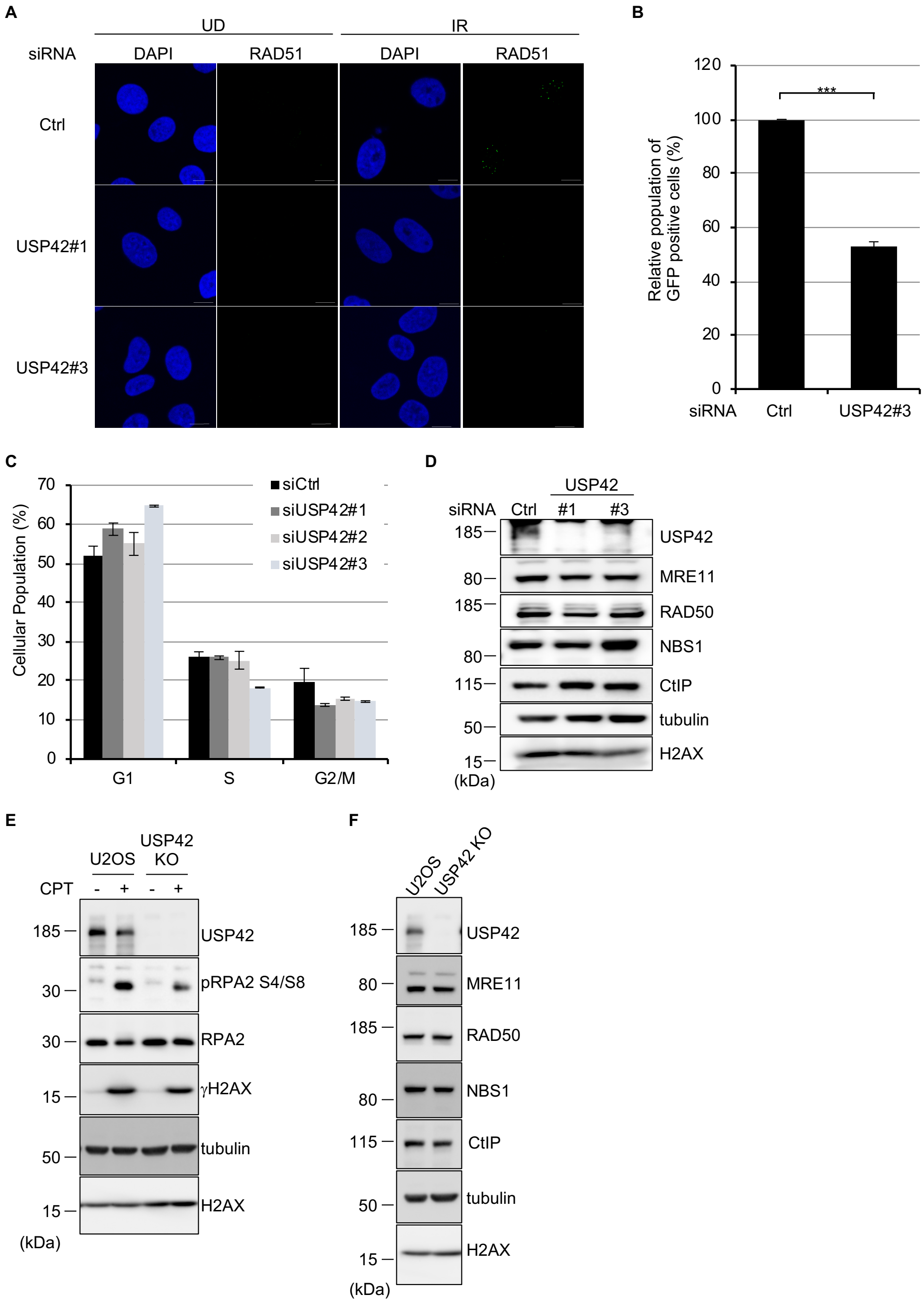

Supplementary Figure S2 USP42 promotes HR by facilitating DNA-end resection

(A) RAD51 foci formation efficiency was examined with the cells transfected with the indicated siRNAs. The Representative images are shown. Scale bar: 10 μm. (B) A DR-GFP assay was performed with the indicated siRNAs (mean ± SEM, n=3). (C) U2OS cells were transfected with the indicated siRNAs and then cell cycle profiles were analysed (mean ± SEM,

n=3). (D) U2OS cells transfected with the indicated siRNAs were subjected to immunoblotting analysis with the indicated antibodies. (E) U2OS and USP42 KO cells were treated with CPT and analysed by immunoblotting with the indicated antibodies. (F) U2OS and USP42 KO cells were investigated for the expression of HR factors by immunoblotting with the indicated antibodies. ***: $p < 0.005$.

Figure S3

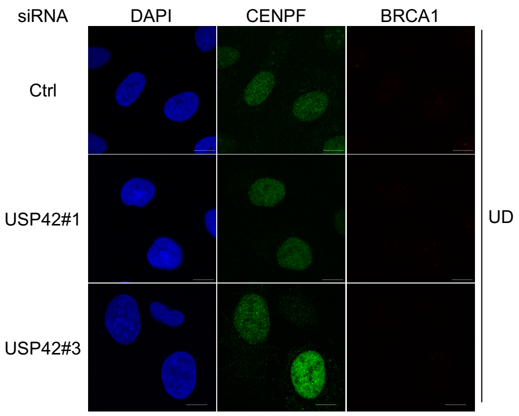

Supplementary Figure S3 USP42 is required for efficient recruitment of BRCA1 to DSB sites

BRCA1 foci formation efficiency was examined with the cells transfected with the indicated siRNAs. Representative images for undamaged cells (UD) are shown. Scale bar: 10 μm.

Figure S4

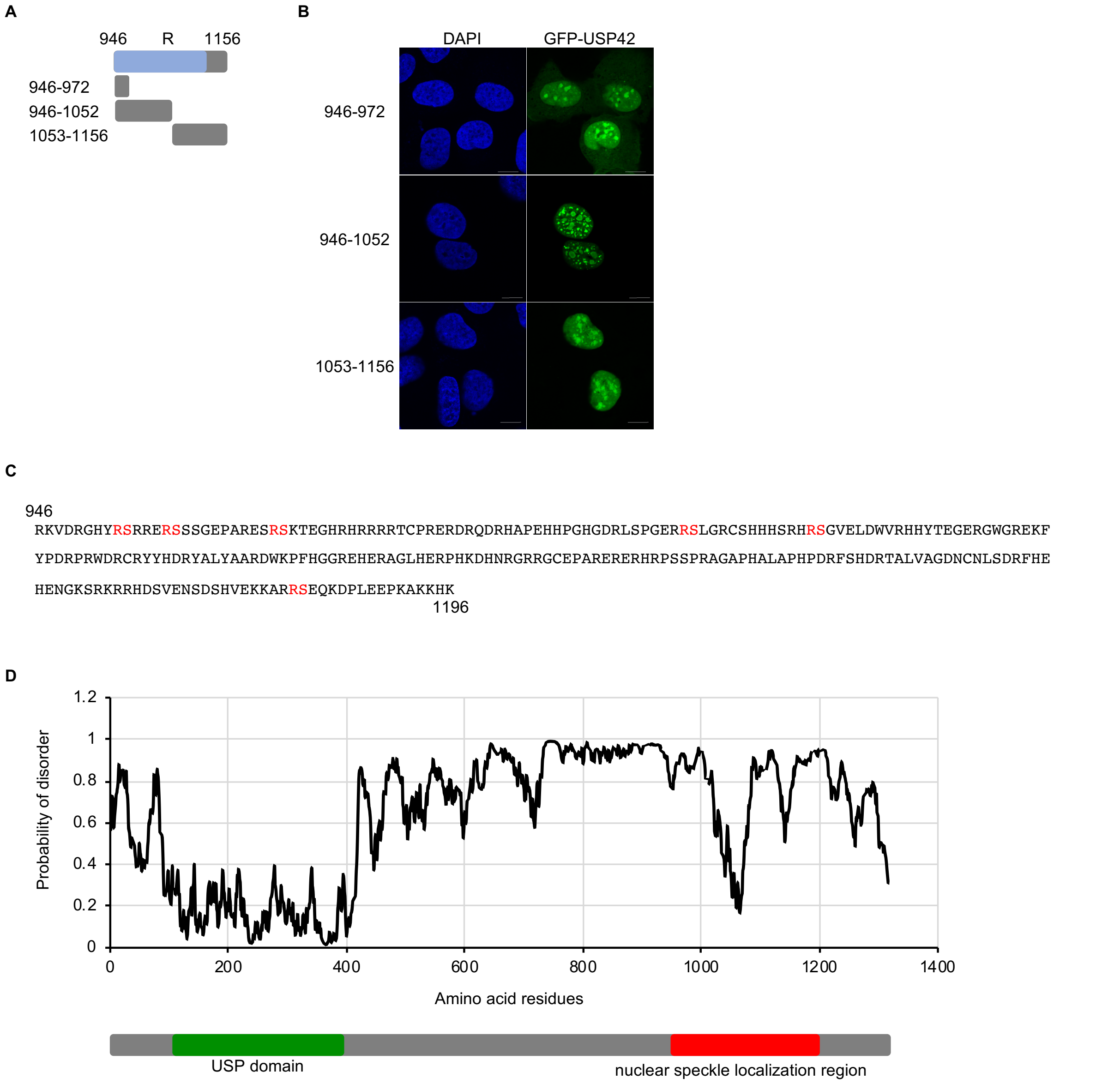

Supplementary Figure S4 Nuclear speckle localization of USP42 is required for efficient homologous recombination

(A) Schematic representation of truncated USP42 proteins. The numbers represent amino acid residues. R: arginine-rich region. (B) Subcellular localization of GFP-fused truncated USP42 proteins. Cells transfected with the plasmids coding the indicated GFP-USP42 were subjected to fluorescence microscopy analysis. Scale bar: 10 μm. (C) Amino acid sequence of USP42 (946-1196 a. a.) in which RS repeats are indicated with red characters. The numbers represent amino acid residues. (D) The probability of disorder of USP42 protein predicted by IUPred2A

was plotted against amino acid residues. A schematic representation of USP42 is also shown underneath the graph. The USP domain (green) and nuclear speckle localization region (red) are indicated.

Figure S5

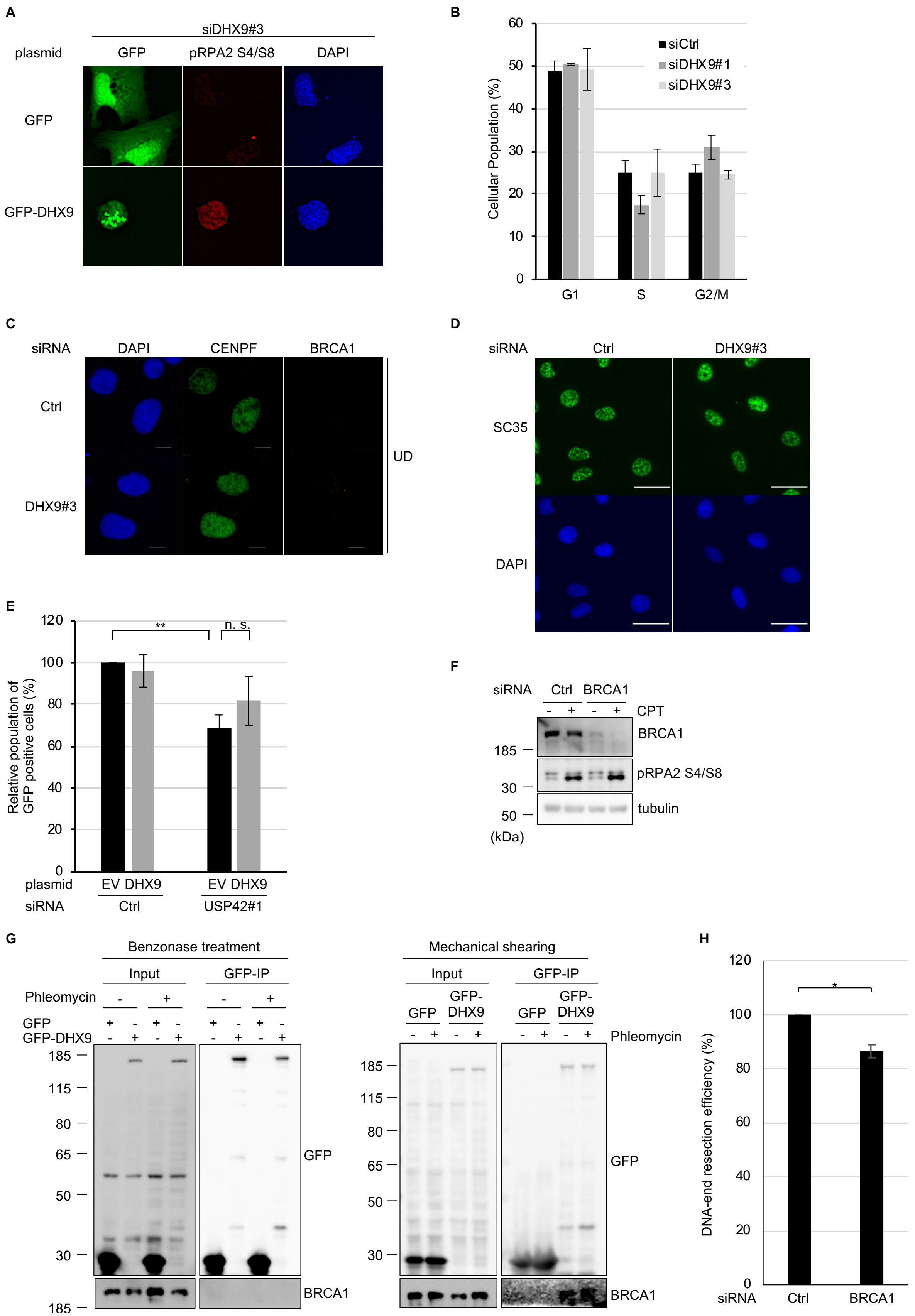
A
siDHX9#3
plasmid
GFP
pRPA2 S4/S8
DAPI
GFP
GFP-DHX9
B
Cellular Population (%)
siCtrl
siDHX9#1
siDHX9#3
G1
S
G2/M
C
siRNA
DAPI
CENPF
BRCA1
Ctrl
DHX9#3
UD
D
siRNA
Ctrl
DHX9#3
SC35
DAPI
E
Relative population of GFP positive cells (%)
**
n. s.
plasmid
EV DHX9
EV DHX9
siRNA
Ctrl
USP42#1
F
siRNA
Ctrl
BRCA1
CPT
BRCA1
pRPA2 S4/S8
tubulin
(kDa)
G
Benzonase treatment
Input
GFP-IP
Phleomycin
GFP
GFP-DHX9
GFP
BRCA1
Mechanical shearing
Input
GFP-IP
GFP
GFP-DHX9
Phleomycin
GFP
BRCA1
H
DNA-end resection efficiency (%)
*
siRNA
Ctrl
BRCA1

Supplementary Figure S5 USP42 is epistatic with DHX9 in the cellular survival after DSB induction and promotes resolution of DSB-induced R-loop.

(A) U2OS cells that were transfected with the siRNA (siDHX9#3) and plasmid coding either GFP or siRNA-resistant GFP-DHX9 were treated with 1 μM CPT and then analysed by immunofluorescent staining with an anti-pRPA2 S4/S8 antibody. Cells transfected with plasmids were detected by GFP signal. (B) U2OS cells were transfected with the indicated siRNAs, and then cell cycle profiles were analysed (mean ± SEM, n=3). (C) BRCA1 foci formation efficiency was examined with the cells transfected with the indicated siRNAs. Representative images for undamaged cells (UD) are shown. Scale bar: 10 μm. (D) U2OS cells transfected with the indicated siRNA were subjected to immunofluorescent staining with an anti-SC35 antibody. Scale bar: 40 μm. (E) A DR-GFP assay was performed with the cells transfected with the indicated siRNA and plasmid (mean ± SEM, n=4). EV: empty vector, DHX9: mCherry-DHX9. (F) U2OS cells transfected with the indicated siRNAs were treated with CPT and analysed by immunoblotting with the indicated antibodies. (G) U2OS cells transfected either with GFP or GFP-DHX9 coding plasmid were treated with phleomycin. Cells were lysed either with the IP lysis buffer containing 150 mM NaCl (left) or by mechanical shearing (right). The interaction between DHX9 and BRCA1 was analysed by immunoprecipitation with an anti-GFP antibody followed by immunoblotting with the indicated antibodies. (H) A DNA-end resection assay was carried out with the cells transfected with the indicated siRNAs (mean ± SEM, n=3). *: $p<0.05$, **: $p<0.01$, n. s.: not significant.

Supplementary Table S1 The siRNAs used in this research

Target	siRNA#	Sequence
AAAS	1	GAGCAAGUGUGGAAGAGAU(dTdT)
	2	CCACACCAACAAGUUUGCA(dTdT)
ACF	1	GCAACCCUAAUUAACCUGA(dTdT)
	2	CAAGACUUAGCAGCAUAUA(dTdT)
ACIN1	1	CAGAAGAAACCUUCCAUCA(dTdT)
	2	CACAUCACCUAUCCCUACA(dTdT)
ACTL6A	1	GUAUACUCAGGAAAAGAAU(dTdT)
	2	GAGGAAACACACUAAUACA(dTdT)
AKAP8	1	GACAGUAGUGCACUUGGAA(dTdT)
	2	GUUUCAAGCUUCAGCCUUU(dTdT)
AQR	1	CAGUCAAAAAGUCUGAGUA(dTdT)
	2	AGUUCACUCAUACACAGAU(dTdT)
ARNTL	1	GAGAACCCAGGUUAUCCAU(dTdT)
	2	GAUCAAGUAGUCCCAGUAA(dTdT)
ASH2L	1	GUAUGAACGGGUUUUGUUA(dTdT)
	2	CUGAGAACACCUGAAAUCA(dTdT)
ATXN1	1	CUGAGAAUGGCGAACUGAA(dTdT)
	2	GAGACUGGUGCAUAUGCUU(dTdT)
BCAS2	1	CCAGUUAGAGCAUCAAGCA(dTdT)
	2	CAGAAGGAACUUCAGAAGU(dTdT)
BCLAF1	1	CGAAGGUAGCUCUAUGGUU(dTdT)
	2	ACAUCGUAGGGUGACUGAU(dTdT)
BRCA1	-	GGAACCUGUCUCCACAAAG(dTdT)
BUD31	1	GAGAUCUUUCUGCUGUCUA(dTdT)
	2	CGAGAGAUCUUUCUGCUGU(dTdT)
C12orf11	1	GAGGAAGAGUUACAGACUA(dTdT)
	2	CAGUGAAGUUCAUAGUGUU(dTdT)
C14orf92	1	AGUGCUUUUGGGUCCUAGA(dTdT)
	2	CACUCUAUAGGAACUCAGU(dTdT)
C21orf66	1	GUAAUGAGAUGACUCCCGU(dTdT)
	2	GUAAAGGCCGCCUUGUUAG(dTdT)
CCAR1	1	GAGAAGUAGAGUCCUUAGA(dTdT)
	2	CUCAGAUCGAUGCUAUAGA(dTdT)
CEBPA	1	CUCCAAUGCCUACUGAGUA(dTdT)
	2	CGAAGCACGAUCAGUCCAU(dTdT)
CEBPB	1	CUGAGUAAUCGCUUAAAGA(dTdT)
	2	CGUCUGUAUAUUUUGGGAA(dTdT)
CHERP	1	CCAAUGUGCCCUACUUCGA(dTdT)
	2	GAUAAGUGGGACCAGUAUA(dTdT)
CLSTN1	1	GGAUCCCUCAACUGGACCA(dTdT)
	2	GAGUUGAGGCUCUCCAUCU(dTdT)
CRSP6	1	AGUUGAGACACCUAGUGUA(dTdT)
	2	UGAUGUCCAAAUUCCUAGU(dTdT)
CtIP	-	GCUAAAACAGGAACGAAUC(dTdT)
DDX17	1	CUCUAGCCAGCAGUUUAGU(dTdT)
	2	GUGGUUAUUUGGAUUGCUU(dTdT)
DDX3X	1	GUGUCAAUUCUGCUCUACA(dTdT)
	2	CUCAGAUUCGUAGAAUAGU(dTdT)
DDX46	1	CAGAGAAUCAGUGAAUACU(dTdT)
	2	CUCUGAAGCCGCAAUUACA(dTdT)
DDX48	1	GAGCAAUCAAGCAGAUCAU(dTdT)
	2	GACUACAUGAAUGUCCAGU(dTdT)
DDX5	1	UGAAGCAGAUAGAAUGCUU(dTdT)
	2	CGACCUUAUCUCUGUGCUU(dTdT)
DHX9	1	CUGCUAUCAUCAGCCAGUU(dTdT)
	2	CAGGAUAUGUGCAGGUGUU(dTdT)
	3	GUAAAUGAACGUAUGCUGA(dTdT)
DNAJA1	1	GAAAGAUGGCCAGAAGAUA(dTdT)
	2	GAGUGAAGGACUGUAAUCA(dTdT)
EIF4A1	1	CAGGUUUCUUUAGUCAUCA(dTdT)
	2	GACGUGAUUAUGAGGGAGU(dTdT)
ELAVL1	1	CUGAUGAAUUCUCCCUUGU(dTdT)
	2	GUGAUCAAAGACGCCAACU(dTdT)
EWSR1	1	GACUCUGACAACAGUGCAA(dTdT)
	2	CCAUGAUCCACAUCUACCU(dTdT)
EXOSC9	1	GAAAGAUCGGGUUCCGUUU(dTdT)
	2	GAGCUAAUAUUGAAAGCUU(dTdT)
FAM3C	1	GUGCAUGAGUAUUGUCUCU(dTdT)
	2	GCACUUAAAUUUCGAACCU(dTdT)
FUS	1	GAGUCUGUGGCUGAUUACU(dTdT)
	2	GAGGUAACUAUGGCCAAGA(dTdT)

Supplementary Table S2 The antibodies used in this research

Antibodies	SOURCE	IDENTIFIER
Anti-WDR5 antibody	Abcam	Cat#ab56919
Anti-RNA Helicase A antibody	Abcam	Cat#ab54593
GTF3C2 Antibody	Proteintech	Cat#27494-1-AP
Anti-USP42 antibody produced in rabbit	ATLAS	Cat#HPA006752
Anti-USP42 antibody	Abcam	Cat#ab121254
Monoclonal Anti-splicing Factor SC35 antibody produced in mouse	Sigma-Aldrich	Cat#S4045
Anti-SC35 antibody-Nuclear Speckle Marker	Abcam	Cat#ab11826
Anti-RPA32/RPA2 (phosphor S4+S8) antibody	Abcam	Cat#ab87277
Anti-phospho-Histone H2A.X (Ser139) antibody, clone JBW301	Merck Millipore	Cat#05-636
Monoclonal Anti-a-Tubulin antibody produced in mouse	Sigma-Aldrich	Cat#T9026
Anti-BrdU Antibody	GE Healthcare	Cat#RPN202
Anti-RAD51 Antibody	Bio Academica	Cat#70-001
BRCA1 antibody (D-9)	Santa Cruz Biotechnology	Cat#sc-6954
Anti-53BP1 Antibody, clone BP13	Merck Millipore	Cat#MAB3802
Anti-RPA32/RPA2 antibody [9H8]	Abcam	Cat#ab2175
Anti-Histone H2A.X antibody-ChIP Grade	Abcam	Cat#ab11175
Cyclin A (H-432) Antibody	Santa Cruz Biotechnology	Cat#sc-751
Mre11 antibody [12D7]	Gene Tex	Cat#GTX70212
Rad50 antibody [13B3]	Gene Tex	Cat#GTX70228
Anti-p95/NBS1 antibody	Abcam	Cat#ab23996
CtIP antibody (mAb)	Active Motif	Cat#61141
Anti-CENPF antibody	Abcam	Cat#ab5
Anti-GFP	Roche	Cat#11814460001
Ubiquitin (P4D1) Mouse mAb	Cell Signaling TECHNOLOGY	Cat#3936S
Anti-HA (12CA5)	Roche	Cat#11583816001
Anti-DNA-RNA Hybrid Antibody, clone S9.6	Merck Millipore	Cat#MABE1095

Supplementary Table S3 List of USP42 interactors

Gene symbol	Accession number	Number of matches
DSP	P15924	91
PRPF8	Q6P2Q9	89
USP42	Q9H9J4	87
EFTUD2	Q15029	67
SNRNP200	O75643	60
KPRP	Q5T749	58
KRT6B	P04259	40
PRKDC	P78527	39
DHX9	Q08211	38
IGHV3-72	A0A0B4J1Y9	33
LRPPRC	P42704	32
PRPF6	O94906	28
VIM	P08670	24
HNRNPM	P52272	24
SRSF6	Q13247	24
RPS3	P23396	23
MYH6	P13533	22
RPS4X	P62701	22
MATR3	P43243	21
EIF4A3	P38919	21
SRSF1	Q07955	20
RPL7	P18124	19
RPS9	P46781	19
RPS18	P62269	18
SAP18	O00422	18
SLC25A6	P12236	18
RPL7A	P62424	17
DDX3X	O00571	16
ARG1	P05089	16
PARP1	P09874	16
SLC25A4	P12235	16
CRNKL1	Q9BZJ0	16
PRPF19	Q9UMS4	16
EIF3CL	B5ME19	15
MYO1C	O00159	15
HNRNPC	P07910	15
PIP	P12273	15
CDC5L	Q99459	15
SF3B1	O75533	14
TUBA4A	P68366	14
TUBA3C	Q13748	14
ACIN1	Q9UKV3	14
ILF3	Q12906	13
TUBB2A	Q13885	13
SF3B3	Q15393	13
SRSF7	Q16629	13
